# Extracellular Vesicles and Exosomes: Insights From Exercise Science

**DOI:** 10.3389/fphys.2020.604274

**Published:** 2021-02-01

**Authors:** Joshua P. Nederveen, Geoffrey Warnier, Alessia Di Carlo, Mats I. Nilsson, Mark A. Tarnopolsky

**Affiliations:** ^1^Department of Pediatrics, McMaster University Medical Centre (MUMC), Hamilton, ON, Canada; ^2^Institut of Neuroscience, UCLouvain, Université catholique de Louvain, Ottignies-Louvain-la-Neuve, Belgium; ^3^Exerkine Corporation, McMaster University Medical Centre (MUMC), Hamilton, ON, Canada

**Keywords:** resistance exercise, aerobic exercise, exosome, extracellular vesicle, EV isolation, size-exclusion chromatography, circulation, exerkine

## Abstract

The benefits of exercise on health and longevity are well-established, and evidence suggests that these effects are partially driven by a spectrum of bioactive molecules released into circulation during exercise (e.g., exercise factors or ‘exerkines’). Recently, extracellular vesicles (EVs), including microvesicles (MVs) and exosomes or exosome-like vesicles (ELVs), were shown to be secreted concomitantly with exerkines. These EVs have therefore been proposed to act as cargo carriers or ‘mediators’ of intercellular communication. Given these findings, there has been a rapidly growing interest in the role of EVs in the multi-systemic, adaptive response to exercise. This review aims to summarize our current understanding of the effects of exercise on MVs and ELVs, examine their role in the exercise response and long-term adaptations, and highlight the main methodological hurdles related to blood collection, purification, and characterization of ELVs.

## Introduction

Over the last 60 years, the study of exercise science has yielded the immutable fact that habitual exercise confers remarkable health benefits, decelerates biological aging, and prolongs lifespan. There are a wealth of original studies, reviews and meta-analyses demonstrating the beneficial effects of physical activity and exercise across all organ systems in humans, thus protecting against a diverse spectrum of disease states. Although these benefits are most evident in organs directly involved in movement, respiration, and blood-flow (e.g., musculoskeletal, cardiorespiratory, and nervous), positive effects may also be seen in less obvious systems (e.g., integumentary, reproductive, and digestive).

The provision of these remarkable health benefits is obviously complex and multi-factorial (Warburton et al., 2006; Pedersen and Saltin, 2015), but likely partly attributed to the myriad of bioactive molecules released into circulation during exercise, collectively termed exercise factors or ‘exerkines.’ The pioneering work of Pedersen et al. (2001, 2003) identified interleukin 6 (IL-6) as the first muscle-derived exerkine released by skeletal muscle, and thus was classified as a myokine. Currently, over ∼300 exercise factors have been identified, many of which indeed appear to be contractile activity-regulated (Le Bihan et al., 2012; Raschke et al., 2013; Hartwig et al., 2014). Skeletal muscle makes up ∼40% of total bodyweight and possesses the capacity to act as an endocrine organ, particularly during exercise; however, any tissue/cell type capable of secretion may theoretically add to the global ‘exercise secretome,’ including adipose tissue, liver, lymphocytes, endothelial cells, and platelets. Outside of the classical peptide secretion pathway, relatively little is known about how these factors are transported in circulation from their tissue of origin to nearby or distant targets to exert their biological effects. However, a rapidly growing area of research pertaining to extracellular vesicles (EVs) has begun to uncover a potential delivery mechanism, with some data suggesting that specific EV sub-populations transport diverse types of cargoes, including various RNA species, proteins, and metabolites. While much remains to be elucidated on the role of EVs in mediating cell-to-cell communication, organ cross-talk, and in the adaptive response to exercise, a significant body of knowledge has accumulated over the last decade.

This review will provide an overview of the main EV sub-populations, with a specific focus on exosomes as exerkine transporters and mediators of the exercise response. Thereafter we summarize the current understanding of the effects of exercise on microvesicles and exosomes and conclude by addressing the major methodological hurdles associated with blood collection, purification and characterization of exosome-like EVs (ELVs).

## Overview of Extracellular Vesicle Biology

Intercellular communication is a crucial physiological function in multi-cellular organisms essential for the sharing of both signals and resources (Raposo and Stoorvogel, 2013). This communication is a dynamic process that allows the body to carry out necessary functions as well as maintain homeostasis. While gap junctions and synapses allow for the propagation of signals via direct cell-to-cell communication that occurs due to their close physical proximity (Jabeen and Thirumalai, 2018), systemic signals rely on an alternative pathway of communication such as those based on receptor-ligand interactions occurring on cell membranes.

Extracellular vesicles are emerging as another mechanism of intercellular communication through the release or shedding of vesicles by secretory cells. EVs are lipid membrane-enclosed vesicular structures, which are purported to carry a variety of cellular cargo (Ha et al., 2016), such as lipids (Dang et al., 2017), nucleic acids (Ridder et al., 2014), and proteins (Doyle and Wang, 2019). It is hypothesized that these cargoes are transported to both local and distant recipient cells, where they can exert an influence upon recipient cell function in a juxtacrine and endocrine manner, respectively.

Currently, there are three primary classifications of extracellular vesicles; apoptotic bodies, microvesicles, and exosomes (El Andaloussi et al., 2013). These EV subtypes are differentiated by both their size and the nature of their biogenesis (Yáñez-Mó et al., 2015), though there is some overlap ultimately leading to some confusion about the nomenclature (Gould and Raposo, 2013). Given the lack of specific markers for each of the aforementioned EV subpopulations, the International Society of Extracellular Vesicles (ISEV) has suggested the generic term “EVs” for the vesicles released from the cell (Théry et al., 2018), with some classification based on size.

Apoptotic bodies are the largest of the extracellular vesicles (∼500–4000 nm) that are formed as a consequence of programmed cell death (Elmore, 2007). A cell undergoing apoptosis progresses through a number of stages, culminating in the destruction of cellular content enclosed in distinct membrane-bound vesicles, termed apoptotic bodies (Kerr et al., 1972; Bao and Shi, 2007), and thus are often characterized by the presence of organelles and/or nuclear content in their lumen. While not typically associated with extracellular vesicles, unconventional secretion processes such as lysosome vesicle secretion and/or secretory autophagy may release numerous cytoplasmic substrates into the extracellular environment under various conditions (Spaulding et al., 2018), possibly through similar but different mechanisms as other EVs (Ponpuak et al., 2015; Gudbergsson and Johnsen, 2019).

Microvesicles (MV), also known as ectosomes, microparticles, or shedding vesicles, are categorized by their size of ∼100–1,000 nm and form directly from the outward budding of the plasma membrane (Cocucci et al., 2009). MVs are differentiated from apoptotic bodies by size, but also their formation, content and membrane-specific antigens, as they originate from the plasma membrane. Given the overlap in size, the direct outward budding and fission of the plasma membrane distinct to MV formation has traditionally been the primary distinguishing factor between MV and exosomes (Akers et al., 2013), though evidence suggests that ELVs can also be released via budding as well (Booth et al., 2006).

Exosomes are the smallest of the vesicles, measuring ∼40–120 nm and undergo a complex process that involves inward budding of endosomes (French et al., 2017). The studies by Harding et al. (1983) and Pan and Johnstone (1983), published at the same time, observed the release of small EVs into the extracellular space during the maturation of reticulocytes. The process of vesicular secretion was determined to be similar to reverse endocytosis, and thus the small extruded vesicles were identified and subsequently termed “exosomes” (Johnstone et al., 1987).

Since their discovery, extensive research has been conducted, but the biology of exosomes is still not fully understood. Over the last two decades, there has been an accelerated interest in exosome research because of their putative role as mediators of intercellular communication, with relevance to pathophysiology, diagnostics, drug delivery, and discovery of new therapeutic compounds (Akers et al., 2013; Lässer, 2015; Edgar, 2016).

**FIGURE 1:**
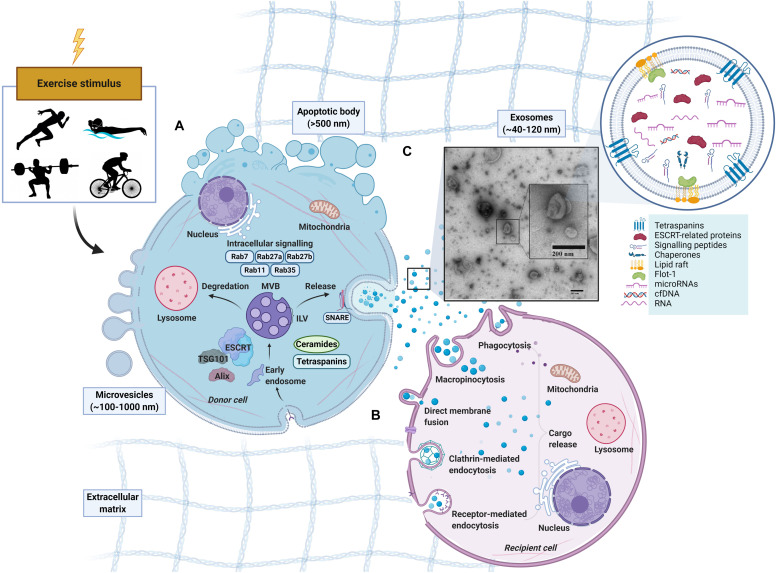
Schematic representation of extracellular vesicles released by cells in response to exercise. Apoptotic bodies (∼>500 nm), microvesicles (∼100–1,000 nm) and exosomes (∼40–120 nm). **(A)** Exosome formation. Early endosomes form from the inward budding of the plasma membrane (PM) before maturing into late endosomes which contain multiple intraluminal vesicles (ILVs). With the presence of ILVs, the late endosome can also be characterized as a multivesicular body (MVB) (Sotelo and Porter, 1959; Akers et al., 2013). MVBs exist in different subpopulations (White et al., 2006) can either fuse with a lysosome for degradation, or fuse with the PM to release ILVs into the extracellular space as exosomes (Akers et al., 2013; Crenshaw et al., 2018). MVB development in an Endosomal Sorting Complex Required for Transport (ESCRT)-dependent or independent manner with tetraspanins (Hemler, 2005; Pols and Klumperman, 2009) or ceramides (Trajkovic et al., 2008) being involved. Rab GTPases (RABs) and soluble *N*-ethylmaleimide sensitive factor attachment protein receptors (SNAREs) may also regulate the cargo sorting and transport of exosomes. **(B)** Exosome uptake in recipient cell. Following release, exosomes can be taken up by recipient cells by direct binding to plasma membrane or engulfment (French et al., 2017). The uptake of exosomes and EVs can be through several avenues including endocytosis (via phagocytosis, micropinocytosis, clathrin-, caveolin-, and/or receptor-mediated endocytosis) or fusing directing to the plasma membrane of the recipient cell (Skog et al., 2008; Lässer et al., 2011; Vallhov et al., 2011). After binding, the cargo from the donor-cell exosome is released. **(C)** Exosome-like vesicles (ELV) isolated via SEC from human platelet-free plasma, visualized by electron microscopy.

The process of exosome biogenesis stems from the endocytic pathway, a process that results in the internalization of cellular materials and/or extracellular ligands, directing them to lysosomes or cell-surface membranes (Figure 1). While covered extensively elsewhere (Raposo and Stoorvogel, 2013; Lässer, 2015; Yáñez-Mó et al., 2015; Edgar, 2016), the process of exosome formation is important in the context of their identification, as the involved proteins are often used as markers for the definition of exosomes. Indeed, due to their enrichment and involvement during exosomes formation, the tetraspanins (e.g., CD9, CD81, and CD63) in addition to the tumor susceptibility gene 101 (TSG101) and ALG2 interacting protein X (Alix) have been used as positive markers for exosomes. However, there is not a single surface marker that specifically defines them (Barile and Vassalli, 2017). In fact, over 100 proteins have been listed as potential exosome biomarkers (Keerthikumar et al., 2015) and include multivesicular body formation proteins (i.e., Alix), chaperones (i.e., heat shock proteins), lipid rafts (i.e., flotillin), vesicle adhesion (i.e., tetraspanins) and membrane trafficking proteins such as Rab proteins. Once MVBs mature and are sorted by either pathway, Rab GTPases (e.g., Rab11, Rab27, and Rab35) regulate vesicular trafficking of MVBs toward the plasma membrane and ultimately assist in the secretion of exosomes (Stenmark, 2009; Pfeffer, 2010, 2013; Blanc and Vidal, 2018), possibly through soluble *N*-ethylmaleimide-sensitive factor attachment protein receptors (SNAREs) (Bonifacino and Glick, 2004; Hessvik and Llorente, 2018). Following secretion from the donor cell, the exosomes fuse with the plasma membrane and/or are taken up by the cell of origin or recipient cells, where their cargo is released (for representative schematic, refer to Figure 1). The surface molecules expressed on the membrane of the exosome likely play a role in determining the uptake mechanism utilized (Mulcahy et al., 2014).

Exosomes have been isolated *in vivo* in numerous bodily fluids such as serum, plasma, saliva, urine, breast milk, cerebrospinal fluid, semen, and amniotic fluid (Keller et al., 2003). Indeed, it appears that nearly all cell populations secrete EVs that are distinct to the cell of origin and is a conserved biological process (Deatherage and Cookson, 2012; Schwechheimer et al., 2013; Woith et al., 2019). Due to the residency of exosomes in a variety of bodily fluids, it has been proposed that they possess some prospective involvement in certain physiological and pathological processes (Admyre et al., 2003; Bard et al., 2004; Caby et al., 2005; Lässer, 2015; Ha et al., 2016). Several functions of exosomes have been reported including the regulation of immune response (Raposo et al., 1996; O’Neill and Quah, 2008), presentation of antigens (Raposo et al., 1996; Admyre et al., 2003), angiogenesis (Fan, 2014), carriage of proteins and RNAs (e.g., mRNA and microRNA) (Ratajczak et al., 2006; Valadi et al., 2007), transfer of infectious particles (e.g., prions or viruses) (Nguyen et al., 2003; Fevrier et al., 2004), and cell-to-cell communication. Ultimately, over ∼400 proteins have been previously established to be present in ELVs or MVs derived from skeletal muscle cells alone (Forterre et al., 2014a; Aswad et al., 2014). Thus, the emerging importance of exosomes as cargo carriers for intercellular communication and systemic signaling make them excellent targets for exploration of circulating factors that may underlie the benefits to exercise across all organ systems.

## Extracellular Vesicles – Potential Mediators of the Multi-Systemic Exercise Response?

The long-term adaptations to exercise include significant health benefits and protects against a variety of chronic diseases (Kruk, 2009; Pedersen and Saltin, 2015). While low-intensity exercise is sufficient to improve overall health, training benefits are usually dose-dependent with reductions in mortality risk greater at higher intensities (Hupin et al., 2017). The combined weight of the evidence suggests that exercise exerts its multi-systemic effects by facilitating juxtracrine, autocrine, and paracrine communication between cells. Exercise also appears to promote cross-talk between tissues/organs that are not located in a close spatial proximity (i.e., endocrine signaling). While the underlying mechanisms of this systemic benefit are complex and multifactorial (Warburton et al., 2006; Pedersen and Saltin, 2015), the fact that skeletal muscle accounts for ∼40% of the human bodyweight, changes its metabolic profile dramatically during exercise, and is an endocrine organ capable of secretion, underscores it as a likely origin of therapeutic factors. These muscle-derived factors, or ‘myokines’ (Pedersen and Febbraio, 2008), have the capability to act in a paracrine, autocrine, and/or endocrine fashion (Pedersen, 2011; Nielsen et al., 2014; Párrizas et al., 2015). For example, the first discovered myokine, that is also the most closely studied, is interleukin-6 (IL-6) (Pedersen et al., 2003). Studies have demonstrated that an acute bout of exercise can induce an increase in the production and the secretion of IL-6 by skeletal muscle (Steensberg et al., 2000; Febbraio and Pedersen, 2002). Once released, IL-6 can mediate local tissues, increasing glucose uptake and fatty acid oxidation in the muscle (Al-Khalili et al., 2006; Carey et al., 2006). It may also function as an endocrine agent, capable of increasing lipolysis of adipocyte tissues (Lyngsø et al., 2002; Van Hall et al., 2003; Petersen et al., 2005). Since being coined, over ∼300 other muscle-derived factors have been ‘discovered,’ including a vast number of pro- and anti-inflammatory cytokines (e.g., IL-1α/β, IL-8, IL-10, and IL-15), with almost half being contractile activity-mediated (Quinn et al., 2002; Nielsen et al., 2008; Le Bihan et al., 2012; Raschke et al., 2013; Hartwig et al., 2014). Among these exerkines, osteocrin (Subbotina et al., 2015), brain-derived neurotrophic factor (BDNF) (Matthews et al., 2009), follistatin-like 1 (FSTL1) (Xi et al., 2016), irisin (Lu et al., 2016) and fibroblast growth factor-21 (FGF-21) (Izumiya et al., 2008), vascular endothelial growth factor (VEGF) (Gustafsson et al., 2001) and myostatin (Hittel et al., 2010) have also been investigated for their therapeutic potential. Ultimately, muscle posses a unique secretory profile within the circulatory milieu, compiled of several hundred cytokines and peptides (Bortoluzzi et al., 2006; Yoon et al., 2009; Figueiredo Neto and Figueiredo, 2016).

However, the benefits from exercise that have been ascribed to systemic growth factors secreted from muscle have, until recently, not been examined from a perspective view other than the classical secretory pathway. In this classical pathway, peptides intended for secretion are typically targeted to the endoplasmic reticulum by a secretory signal sequence at the amino terminus (i.e., hydrophobic residues preceded by a positively charged amino acid) before eventually being secreted into the extracellular space (Théry et al., 2002). However, an additional pathway for peptides and/or other signaling molecules lacking a secretory signal sequence that may be otherwise altered in the extracellular space can be secreted in EVs (Choi et al., 2015). The myogenic origin of some subpopulations of EVs have been probed *in vitro*, with results suggesting that myoblast and myotubes are both capable of release EVs in culture (Romancino et al., 2013; Forterre et al., 2014a, b; Choi et al., 2016), possibly originating from different processes (Romancino et al., 2013). Proteomic analysis performed on ELVs released in the conditioned medium from human myotubes revealed proteins related to intercellular trafficking, plasma membrane development, endocytosis, protein synthesis, and free-radical scavenging (Le Bihan et al., 2012). In line with the concept that EVs are, in part, responsible for cell-to-cell communication, evidence from Forterre et al. (2014a) suggests that EVs released from myotubes could be taken up by myoblasts, subsequently upregulating myogenic differentiation (Forterre et al., 2014a). Work from the same group established that the EVs released from myoblasts and myotubes *in vitro* contained proteins and microRNAs (miRNA) that were specific to the particles and not the cells themselves (Forterre et al., 2014b). The process of selective loading of EVs has been examined an *in vitro* model of muscle atrophy (i.e., dexamethasone application to *in vitro* myotubes). During myotube atrophy, there was a marked decrease in miR-23a abundance in the myotubes concomitant with an increase in miR-23a expression in the isolated EVs, ultimately leading to an altered expression of downstream target genes (Hudson et al., 2014b). Similarly, ELV miRNA content has been shown to be different from that of intracellular miRNA content in myotubes, further suggesting that ELV content packaging is likely a similarly regulated process (Nie et al., 2019). Taken together, these data suggest that skeletal muscle can produce bioactive EVs. Our understanding of how skeletal muscle acts as an endocrine organ has evolved, expanding to include the release of EVs. Less established is our understanding of how these signaling molecules, peptides, miRNA can be secreted into EVs and subsequently shuttled elsewhere in response to exercise.

### Identifying the Contribution of Skeletal Muscle to the EV Pool During Exercise

Extensive work has established skeletal muscle as an endocrine organ, and there is growing evidence to support the notion that muscle can release EVs into the circulatory blood. Intramuscular injection of fluorescently labeled EVs resulted in the appearance of fluorescence in distal and contralateral muscles – reinforcing the notion of paracrine-like action of muscle released EVs (Jalabert et al., 2016). Nevertheless, challenges remain for the detection of skeletal-muscle specific EVs (SkMEVs) in response to exercise, as there are limited methods to label and track SkMEVs within the systemic circulation. Instead, skeletal muscle specific markers have been used as a surrogate for specific SkMEVs labels. Initially described by Guescini et al. (2015), population of SkMEVs were identified using α-sarcoglycan (SGCA), a protein that is highly abundant in skeletal muscle, and subsequently verified from the EVs cargo which was enriched with skeletal muscle-specific microRNA myomir mir-206 (Guescini et al., 2015). In response to an acute bout of endurance exercise (i.e., 45 min at ∼65% of VO_2max_) there was no significant change in the abundance of circulating muscle-specific ‘myomir,’ mir-206 1 h post-exercise (Guescini et al., 2015). In this study, while the high-purity SkMEVs were positive for canonical exosome markers TSG-101 and CD81, they only represented ∼1 to 5% of the total plasma-isolated EV population, putting into question the relative importance of skeletal muscle-derived EVs to the total pool of circulating EVs. These findings may support the use of using microRNAs or specific proteins to confirm EV origins.

The release of non- and ‘myomir’ muscle-specific miRNAs (McCarthy, 2008, 2011) has been shown to occur concomitantly with an increase in EVs following acute exercise bout (D’souza et al., 2018; Lovett et al., 2018; Oliveira et al., 2018). In addition, miRNAs found in circulating plasma are altered with different modalities and/or intensities of exercise including various resistance exercise bouts (Cui et al., 2017), marathon running, long-distance cycling and a maximal exercise test (Uhlemann et al., 2014). More recent work from D’Souza and colleagues utilized a more precise method of ELV isolation (size exclusion chromatography; SEC), to identify miRNA in response to exercise. In response to the exercise bout (high intensity interval cycling exercise, 10 s × 60 s), there was altered timing and magnitude of changes of miRNA expression amongst the various tissue preparations (i.e., skeletal muscle, whole plasma and SEC-derived ELVs), suggesting there is no clear inter-tissue relationship during exercise. Interestingly, training status may influence the change in the miRNA expression within exosome-enriched preparations. Well-trained older men exhibited an altered miRNA expression in response to an acute bout of exercise as compared to their sedentary counterparts (Nair et al., 2020). These findings support the notion that exercise promotes an altered miRNA expression, it is important to note that ELVs have been shown to contain a unique miRNA profile as compared to whole cell free plasma (Cheng et al., 2014). Thus, the complete isolation of ELVs from other EVs becomes paramount. There is growing interest in the topic of SkMEV and proposed EV cargo in response to exercise (Rome et al., 2019; Trovato et al., 2019; Vechetti, 2019; Murphy et al., 2020; Fuller et al., 2020). Given the challenge presented in tracking skeletal muscle-derived ELVs, whether miRNA cargo observed immediately following exercise is derived from the *exercising muscle* remains to be elucidated clearly.

## Exercise and Microvesicles – Summary of Studies

A multitude of studies have been conducted in humans examining the effects of acute exercise on circulating EVs, with a predominant focus on the larger sub population known as MVs (>500 nm). These results are important to consider given the potential for a similar response to exercise as their smaller and unique counterparts (i.e., ELVs). Platelet-derived microvesicles (PMVs) are the most abundant, circulating MV population (Sossdorf et al., 2010, 2011; Headland et al., 2015), and have received considerable attention due to their robust response to exercise (Eichner et al., 2018; Wilhelm et al., 2018). Specifically, in response to maximal incremental cycle ergometry (i.e., a VO_2max_ test), PMVs appear to increase during the post-exercise recovery period (Chen et al., 2010; Chaar et al., 2011). Similarly, cycling performed in the demarcated ‘heavy intensity exercise domain’ (Keir et al., 2015) was also shown to increase in circulating PMVs. Furthermore, cycling performed at ∼85% HR_max_ (Maruyama et al., 2012), ∼80% of anaerobic threshold (Sossdorf et al., 2010, 2011), ∼70% of VO_2max_ (Lansford et al., 2016) and a single bout of high intensity interval exercise (Guiraud et al., 2013) all appear to increase PMVs. Work by Wilhelm et al. (2016) examined PMVs during a 1 h bout of heavy intensity exercise (∼65% VO_2max_), showing that PMVs were elevated during and early in post-exercise recovery (Wilhelm et al., 2016). In contrast, moderate intensity exercise (∼45% VO_2max_) did not elicit the same changes in PMVs, suggesting that PMV release in response to exercise is intensity dependent (Wilhelm et al., 2016). It also would appear that the increase in PMVs is relatively short-lived, with concentrations of blood-borne PMVs stabilizing within ∼30 min of aerobic exercise (Wilhelm et al., 2016). While PMV levels remain elevated for ∼1 to ∼2 h post-exercise recovery (Sossdorf et al., 2010, 2011; Chaar et al., 2011; Wilhelm et al., 2016), the circulating PMV content returns to resting baseline relatively quickly.

The time-course dynamics and response to exercise is less clear in other MV subpopulations. Release of endothelial-derived microvesicles (EMVs) in response to exercise has been well studied, although there is limited consensus in the field likely due to the variety of isolation methods. Studies have reported a post-exercise increase (Kirk et al., 2014; Lansford et al., 2016), no change (Möbius-Winkler et al., 2009; Sossdorf et al., 2010; Chaar et al., 2011; Guiraud et al., 2013) and even decreases in both healthy (Wahl et al., 2014) and obese individuals (Durrer et al., 2015). Similar to their PMV counterparts, these contrasting results may be attributable to varying responses to types of exercise, intensities, or the activity level of the population under study. Indeed, to add another dimension of complexity, training status may change the exercise-induced release of these MVs. Work by Wahl et al. (2014) examined the appearance of varying subsets of EMVs in response to different modalities of ‘heavy intensity exercise’ in highly trained individuals (∼65 mL⋅min^–1^⋅kg^–1^). Three different endurance protocols [∼55% peak power output (PPO) × 130 min; 4 × 4 bouts of 95% PPO; 4 s × 30 s ‘all out’ exercise], were undertaken by highly trained cyclists. In response to all exercise protocols, there was a decrease in circulating EMVs. The authors proposed an improved rate of clearance due to the training status of the individuals, and that avenue represents an exciting frontier for future investigations.

Together, these publications offer an important insight into the release of circulating MVs. Important lessons can be taken from the fact that circulating EV populations of a larger diameter are released in response to exercise, and the training status of the individual may play an important role in uptake of MVs. These subpopulations (e.g., EMVs and PMVs) may be bioactive, capable of interacting with the vascular endothelium and may play a significant role in physiological function and the response to exercise. The reader is further encouraged to refer to previous literature for an excellent review specifically on MV release and exercise (Wilhelm et al., 2018).

Future work will need to continue to address what population of EVs, whether MV or ELVs, the miRNAs are being transported – and their destination. Work from Whitham et al. (2018) utilized proteomic analysis to suggest that following a bout of 1 h cycling exercise (performed in increments of 30 min at 55%, 20 min at 70%, and ∼10 min at 80% of VO_2max_) that increase in circulatory EVs were likely released from skeletal muscle before being taken up by the liver. However, while this study provides some insight into the characterization of the speculative cargo of plasma EVs via ultra-high performance liquid chromatography tandem mass spectrometry analysis, the isolation method for establishing the ‘exosome’ or ELVs populations was unrefined (i.e., low speed centrifugation), ultimately lacked specificity of the tissue and/or cell of origin, and likely contained a large amount of MV-derived material.

## Exercise and Exosomes – Summary of Studies

Although the field is still in its infancy, a few well-controlled studies have attempted to examine the effects of acute exercise on specific ‘exosome-like EVs’ (ELVs) with complete particle characterization and expression analyses. This limited body of high-quality *in vivo* evidence indeed points to an acute exercise effect, with a significant increase in systemic ELVs during and immediately following exercise (Table 1).

**TABLE 1:**
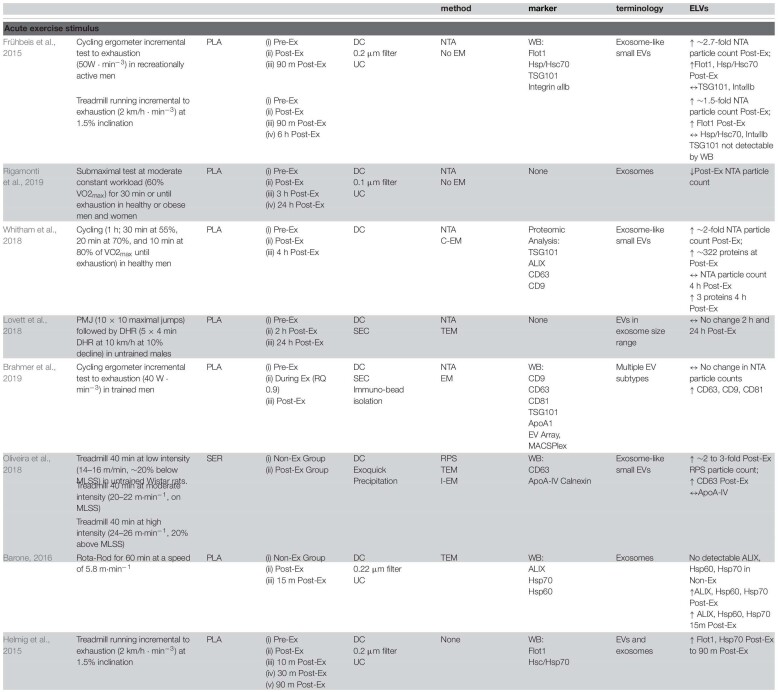
Effect of acute exercise on circulating exosome-like vesicles (ELVs).

*C-EM, cryo-electron microscopy; DC, differential centrifugation; DHR, downhill running; EM, electron microscopy; EV, extracellular Vesicles; I-EM, immuno-electron microscopy; MLSS, maximal lactate steady state; NTA, nanoparticle tracking analysis; PLA, plasma; PMJ, plyometric jumping; RPS, resistive pulse sensing; RQ, respiratory quotient; SEC, size exclusion chromatography; SER, serum; TEM, transmission electron microscopy; UC, ultracentrifugation; WB, Western Blot.*

### Acute Exercise Studies

Frühbeis et al. (2015) examined the dynamics of ELVs (size ∼100–130 nm) in response to short-duration, incremental cycle ergometry and treadmill running in healthy male volunteers (>3 h physical activity/week). Prior to testing, participants had abstained from exercise for 24 h and were instructed to consume a standardized breakfast on the morning of the tests. In the first exercise setting (*n* = 8, ∼41 years), participants completed a graded cycling protocol starting at 50 W, which was increased incrementally with 50 W every 3 min until volitional exhaustion. On the second testing occasion (*n* = 4, age ∼27 years), participants completed a progressive running protocol starting at 6 km/h at 1.5% grade, which was increased with 2 km/h every 3 min until exhaustion. For both tests, platelet-rich plasma (PRP) was prepared from tripotassium EDTA-coagulated venous blood by standard methods, followed by ultracentrifugation for 2 h at 100,000 *g* to enrich ELV fractions. As per ISEV recommendations, nanoparticle tracking (NTA) and immunoblotting were conducted to examine changes in size, concentration, and expression of ELVs (Lotvall et al., 2014; Théry et al., 2018). Compared to pre-exercise, plasma taken immediately following a maximal, incremental cycling test showed a ∼2.7-fold and ∼1.8-fold increase of ELV concentrations as assessed by NTA, with a return to baseline levels within 90 min of rest (*n* = 2, descriptive data). Furthermore, markers such as HSP/Hsc70 and flotillin 1 (Flot1) were elevated immediately after exercise (mean increase ∼5.2-fold), before returning to baseline 90 min post-exercise, while canonical ELV marker TSG101 was increased but statistically unchanged. These findings were similar to a concurrent study by the same group examining the transient kinetics of cell-free DNA utilizing the same isolation methodology and exercise stimulus (Helmig et al., 2015). Interestingly, the incremental treadmill test resulted in a moderate increase of NTA-quantified ELVs (∼1.5-fold) immediately post-exercise, but remained sustained at this level for the following 90 min. Despite a sustained elevation in NTA-detected particle concentrations following treadmill exercise, protein levels of IntαIIb and HSP/Hsc70 were not increased while Flot1 was only elevated immediately post exercise (TSG101 was undetectable). Additionally, both NTA and immunoblotting results confirmed that circulating ELVs returned to baseline levels 6 h post exercise and remained stable for the next 24 h.

In the study, mean vesicle diameters were ∼120 and ∼165 nm in the cycle vs. treadmill tests, respectively (Frühbeis et al., 2015), but given the fact that the upper limit of ELV size is ∼140 nm (Harding et al., 2013), it is likely that the ELV isolates contained small MVs (∼50–200 nm) and/or chylomicrons (75–600 nm). Notably, it is challenging to assess the true proportion of ELVs in ultracentrifuge-derived isolates considering that they contain (i) significant amounts of lipoprotein particles, including chylomicrons, LDL and HDL [which have been shown to be significantly increased with acute exercise (Søndergaard et al., 2014)] and (ii) smaller MV populations, both of which may interfere with NTA data (Mørk et al., 2017). Furthermore, these results indicate that exercise modality may play a role in ELV release. Although speculative, treadmill incremental tests typically activate more muscle groups as compared to standard cycle ergometry (Gleser et al., 1974; Reybrouck et al., 1975), thereby increasing total blood flow (Matsui et al., 1978; Joyner and Casey, 2015) and ultimately a higher maximal oxygen consumption (Secher et al., 1974; Stromme et al., 1977), and it is possible that these factors contributed to the observed differences in ELV kinetics between these exercise modalities.

To further elucidate the kinetics of ELVs during exercise, Frühbeis et al. (2015) also collected PRP samples at each incremental step during the graded cycling test. Although the canonical ESCRT and tetraspanin markers were not assessed *per se*, the authors reported that other ELV-related proteins, such as Flot1, Hsp/HSC70, and Integrin αIIb, increased at ∼9–12 min of cycling (corresponding to a workload of 150–200 W), before plateauing between ∼15 and 20 min (250–300 W), and rapidly returning to baseline levels 10 min post exercise. Importantly, these data provided the first insight into EV kinetics during exercise and indicate that circulating ELVs begin to accumulate prior to the anaerobic/lactate thresholds and the exponential increase in plasma norepinephrine/epinephrine in healthy, male volunteers (Schneider et al., 1992; Weltman et al., 1994).

**FIGURE 2:**
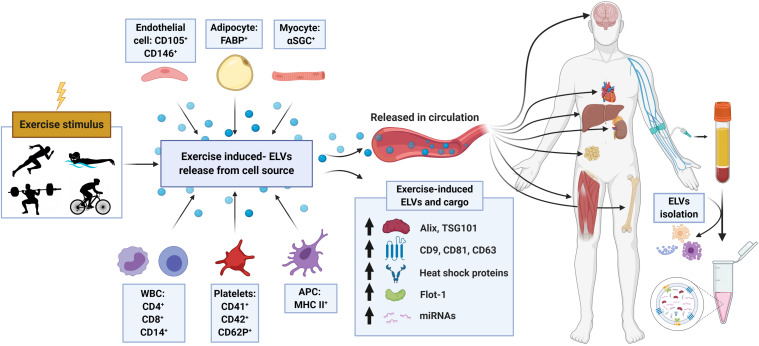
Schematic representation of the proposed origin, release, composition, and cargo of exosome-like vesicles (ELVs) following an exercise stimulus. Following either chronic training or a single bout of exercise, ELVs may be released from muscle or other cell populations and enter into the systemic circulation. Exercise-induced alterations in plasma concentration, exosome markers (e.g., ALIX, TSG101, tetraspanins, flotillin, and heat shock proteins) and cargo (e.g., miRNA, ‘myomiR’ abundance) have been observed. APC, antigen presenting cell; α-SGC, α-sarcoglycan; FABP, fatty-acid-binding proteins; WBC, white blood cells; including monocytes and lymphocytes.

In a follow-up study by the same group (Brahmer et al., 2019), the ELV response to incremental cycling ergometry was assessed by more sophisticated blood collection and EV purification methods in aerobically trained male athletes (∼50 ml⋅kg^–1^ ⋅min^–1^ VO_2max_). Following an overnight’s fast and in a rested state (no exercise for 24 h), participants completed an incremental cycling test starting at 40 W intensity and increased 40 W every 3 min until volitional exhaustion. Venous blood was drawn into tripotassium EDTA-containing tubes at rest, during exercise (at RQ of 0.9), and immediately following exercise, and processed per standard methods to obtain platelet-free plasma (PFP). ELVs were then isolated from PFP using SEC columns and immunobead ‘pull-down’ (e.g., CD9^+^, CD63^+^, CD81^+^ isolation kits), followed by characterization of the exercise response by NTA and immunoblotting. In line with their previous work on UC-derived isolates (Frühbeis et al., 2015) immunoblot analysis of SEC-ELVs revealed an increase in canonical exosome markers during and immediately post-exercise (e.g., CD9, CD63, and CD81). Notably, circulating ELVs, as assessed by immunoblotting, began to increase at submaximal exercise intensities (RQ of 0.9), which confirmed their previous findings on UC-derived isolates (Frühbeis et al., 2015), and appeared to reach peak levels immediately post-exercise. Importantly, NTA analyses were unable to demonstrate this exercise-induced increase in SEC-ELVs, and authors concluded that semi-quantitative methods are more appropriate to use because they are less affected by interference by contaminating factors. In a separate analysis from the same group, Brahmer et al. (2019) demonstrated that exercise-induced SEC-ELVs constitute a heterogenous population, mainly originating from lymphocytes (CD4^+^ and CD8^+^), monocytes (CD14^+^) endothelial cells (CD105^+^ and CD146^+^), and platelets (CD41^+^, CD42^+^, and CD62^+^), and surprisingly, with less contribution from skeletal muscle (e.g., α-sarcoglycan^+^ EVs; SCGA^+^). Together, these findings suggest exercise stimulates ELV release at sub-maximal workloads (i.e., prior to the increase in systemic lactate and spike in catecholamines) but may be potentiated as exercise duration and/or intensity increases. This fairly rapid rate of appearance may suggest that ELVs are primed by early exercise signals, such as shear-stress, mechano-transduction, excitation-contraction coupling, or other Ca^2+^-mediated events while later signals such as hormone release (e.g., cortisol), HPA axis activation (e.g., catecholamines), metabolic stress, and/or reactive oxygen species may potentiate the EV response (Savina et al., 2003; Eldh et al., 2010; Hergenreider et al., 2012). Importantly, ELV release has been shown to be Ca^2+^-mediated (Savina et al., 2003), and genetic knockdown of synaptotagmin-7 (a Ca^2+^ sensor) has been shown to reduce ELV secretion (Hoshino et al., 2013). Following a muscle action potential via the motor neuron, Ca^2+^ is released from the sarcoplasmic reticulum into the local milieu (Kuo and Ehrlich, 2015), ultimately binding to Troponin C to eventually allow for crossbridge cycling. The relatively large flux in Ca^2+^ during skeletal muscle during repeated contractions (i.e., exercise) may result in the accelerated release of ELVs faster than other tissues, but this remains to be elucidated. Together, the rapid appearance of ELVs primarily from cells that are not separated by the blood-tissue barrier puts into question whether skeletal muscle, previously shown to release hundreds of exercise factors, contributes significantly to the exercise-derived ELV pool *during* acute exercise. Instead, the circulatory pool of ELVs observed following exercise may be contributed by a number of cell types (for schematic, refer to Figure 2).

### Effect of Exercise Intensity

Considering that total blood flow and/or number of muscle groups/fibers recruited during exercise may affect the magnitude and/or kinetics of EV release (Frühbeis et al., 2015), the intensity of exercise may also dictate this response. However, there is limited research to support this notion in both animals and humans. For example, Oliveira et al. (2018) exercised rats at low to high exercise intensities and found no differences in systemic serum tetraspanin expression (e.g., CD63) between groups that exercised below or above the maximum lactate steady state (MLSS). However, there was correlative relationship between miRNA content contained in EVs and exercise intensities. ELV isolations were performed on serum fractions using the ExoQuick method (PEG-based), which may not be the optimal for minimizing confounding effects of clotting and avoiding contaminants. Nevertheless, the notion that circulating ELV can be increased by exercise but not necessarily impacted by exercise intensity is supported by this study (Oliveira et al., 2018) and recent human work (Frühbeis et al., 2015).

Furthermore, a relatively high individual variability has been observed in individuals regarding ELV release in response to exercise (Frühbeis et al., 2015; Lovett et al., 2018; Brahmer et al., 2019), which may be related to the methods used to partition the exercise intensity spectrum. Indeed, gold-standard methods for the determination of exercise intensity often rely on fixed-percentages of maximal HR or VO_2max_, which may not correspond to exercise intensity domains (Whipp et al., 2005) and subsequently do not accurately reflect the metabolic stimulus applied. While the concept of ‘responders’ and ‘non-responders’ has been previously proposed in the general exercise physiology field, it remains to be seen whether this phenomenon is based on inaccuracies in assessing exercise workload intensities (Keir et al., 2015; Iannetta et al., 2019). Future work should assess differences in ELV release in response to various exercise intensities in homogenous groups of participants that are matched for gender, age, bodyweight and exercise ability.

### Effect of Health Status

Recent work has attempted to examine the influence of health status on ELV appearance in response to exercise. Rigamonti et al. (2019) examined exercise-induced ELV release in obese- and normal-weight participants. Maximal aerobic capacity was determined via an incremental treadmill walking test to voluntary exhaustion. Following the determination of VO_2max_, participants exercised at a constant workload corresponding to ∼60% VO_2max_ for 30 min or voluntary exhaustion. Plasma ELVs were isolated using differential centrifugation (DC), filtration and ultracentrifugation (UC) and NTA was used to determine ELV size distribution and concentration. The number of ELVs immediately following exercise was significantly lower as compared to resting levels (returning to baseline at 3 h post-exercise), which may be in contrast to previous work on UC-derived ELVs (Frühbeis et al., 2015). Intriguingly, this group also observed that SkM-derived SCGA^+^ EVs (SkMEVs) are elevated in response to exercise, in contrast to other subpopulations such as CD61^+^ EVs (Rigamonti et al., 2019). The determination of ELV in response to exercise becomes challenging when no immunoblot or EM was performed to verify the ELV subpopulation, but this is the first study to observe that ELVs may decrease following acute exercise. Importantly, given that the perturbations in ELV content were no longer observable at 3- and 24 h- post-exercise, this study also reinforces the notion that the rate of appearance (Ra) or disappearance (Rd) of ELVs is a short, temporal one. Furthermore, the authors reported that there may be a sex-based difference in ELV release, with ELVs being lower in females than in their male counterparts, regardless of bodyweight. The influence of sex hormones may therefore play a role in vesicle biogenesis (Toth et al., 2007), and while sex-based differences have been examined in MV populations previously (Durrer et al., 2015; Lansford et al., 2016), there is a paucity of information in literature regarding a purified ELV population.

### Effects of Other Exercise Modalities

While most studies have examined endurance-type exercise modalities, recent work has focused on plyometric-type and/or eccentric contractions specifically. Lovett et al. (2018) examined the impact of eccentric-induced muscle damage on systemic ELV appearance. Participants performed a muscle-damaging exercise protocol that involved plyometric jumping (10 sets × 10 reps at 90% achievable height) and bouts of downhill running at 10% decline at ∼10 km⋅h^–1^ (5 sets × 4 min). ELVs were isolated from blood plasma using size exclusion chromatography (SEC), and TEM and NTA were utilized to verify ELV-enrichment and determine particle concentration. ELV characterization via TEM revealed a size range of ∼30–150 nm, with MV size particles (100–1,000 nm) occurring relatively infrequently, suggesting a relatively purified population (Lobb et al., 2015; Takov et al., 2019). Despite evidence of muscle damage (i.e., a ∼5-fold increase in creatine kinase activity from Pre- to 24 h post exercise), there was no evidence of an increase in ELV number or size at either the 2 or 24 h post-exercise timepoints. The observations by Lovett et al. (2018) are supported by ELV protein expression studies following a non-damaging, combined exercise intervention (Garner et al., 2020). Participants performed a bout of acute aerobic (45 min of two-legged cycle ergometry at 55% of VO_2max_) or combined exercise (aerobic bout followed by single leg knee extensor exercise at 55% of the 1-RM workload until volitional fatigue), and performing skeletal muscle biopsies from the *vastus lateralis* of healthy young men (Garner et al., 2020). In comparing pre- to 1 h post-exercise, Garner et al. (2020) observed no changes in the protein expression of Alix, TSG101 or CD63 in the *vastus lateralis* following either exercise protocols. Aerobic or combined exercise did not increase the expression of most genes associated with exosome biogenesis or release. Though challenging to interpret without complementary data regarding circulating ELVs, these results may be evidence of either a lack of exercise induced ELV release or a rapid replenishment of skeletal muscle derived ELVs by 1 h post-exercise. Given this and other circulatory data in humans (Frühbeis et al., 2015), it would appear that both circulating and ‘muscle resident’ ELVs return to a basal state as early as ∼1 h post-exercise recovery – though much more work is needed.

### Chronic Exercise Studies

A relatively small number of studies have examined or attempted to address exosome-like EV in response to chronic training (Table 2). Bei et al. (2017) found that following 3-weeks of forced swimming in C57BL/6 mice, circulating ELV were increased by ∼1.85-fold as determined by NTA. In the training protocol, forced swimming time was increased until the mice were swimming 90 min twice per day, with serum being compared between the trained group and a sedentary control group. ELVs were isolated via the commercial Exoquick^TM^ method and verified with nanoparticle tracking analysis (NTA), transmission electron microscopy (TEM), and CD63 protein content. Interestingly, following 3 weeks of swim training, there was no observed change in the size of the ELVs, while the number was increased significantly and facilitated a cardioprotective effect against an acute ischemia/reperfusion injury *in vivo*. Work in *db*/*db* mice, a model of type 2 diabetes, would suggest that there is an increase in the number of ELV particles in circulation following chronic training, which may provide some protective effects against disease. Chaturvedi et al. (2015) exercised *db*/*db* and control *db*/*^+^* animals using an 8-week treadmill training model and utilized DC/UC and filtration in order to isolate ELVs from both serum and heart tissue. Following exercise training, there was a greater CD81 and Flot1 expression in circulation of exercise trained animals as compared to their sedentary cohort in both the control (*db*/^+^) and diabetic animal (*db*/*db*), which was mirrored by flow cytometry results. These findings were concomitant with a greater colocalization of CD81 and Flot-1in the vessel walls of the heart. While these findings are interesting and suggest an increase in ELV content with training, it is also worth noting that these studies (Chaturvedi et al., 2015; Bei et al., 2017) do not report when the last bout of exercise was prior to animal sacrifice, so it is challenging to determine whether this increase in circulating content at baseline is indeed due to training, or a transient increase due to a single bout of exercise. In line with this notion, work from Hou et al. (2019) which controlled for the timeframe of ELV collection following the last exercise bout, showed that 4-weeks of forced swimming in Sprague-Dawley rats resulted in no significant change in ELV concentration as compared to untrained animals. For the 4-week training period, animals were allocated into exercise or sedentary groups, with the exercise group performing 10 min⋅day^–1^ progressed to 90 min⋅day^–1^ (7 d⋅wk^–1^). Plasma ELVs were isolated 24 h following last exercise bout via UC and ExoQuick, and characterized by NTA, EM and immunoblots (CD81and TSG101). Similar results were observed when comparing trained human rowers (∼60–120 min⋅day^–1^, 6 d⋅wk^–1^, >1 year training history) to their sedentary counterparts (Hou et al., 2019). While the concentration and protein markers were unchanged, the isolated plasma ELVs from exercise-trained rats were found to have cardioprotective effects against myocardial ischemia/reperfusion injury, suggesting that ELV properties (i.e., cargo) may be altered in response to training in the absence of overall content changes.

**TABLE 2:**
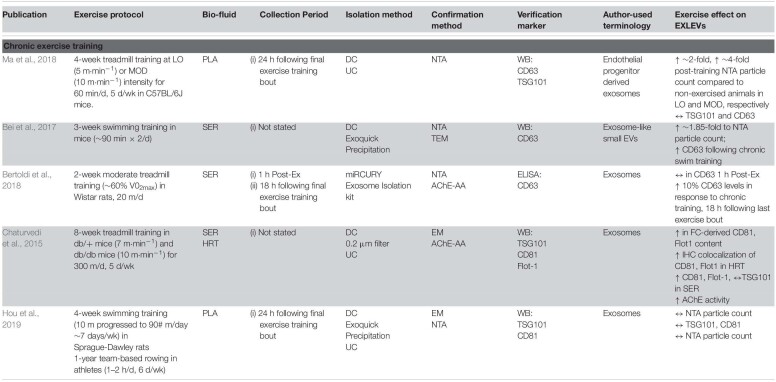
Effect of chronic exercise training on EXLEV.

*AChE-AA, acetylcholinesterase activity assay; DC, differential centrifugation; EM, electron microscopy; ELISA, enzyme-linked immunosorbent assay; FC, flow cytometry; HRT, heart; IHC, immunohistochemistry; NTA, nanoparticle tracking analysis; PES, polyethersulfone filter; PLA; plasma; SER, serum; UC, ultracentrifugation; WB, Western Blot.*

Work from Ma et al. (2018) reinforced the notion that chronic exercise training increases basal endothelial progenitor cell (EPC)-derived ELV content, while at the same time conferring some adaptation to the ELV cargo. For a 4-week training period, C57BL/6J mice were allocated into a sedentary group or exposed to a ‘low intensity exercise’ stimulus or a ‘moderate intensity exercise’ stimulus for 60 min⋅day^–1^ (5 d⋅wk^–1^) with treadmill speed at 5 m⋅min^–1^ for and 10 m⋅min^–1^, respectively (Ma et al., 2018). Heparinized plasma EPC-derived ELVs were isolated 24 h after the last bout of exercise training, via UC. The ELVs were then characterized by NTA and immunoblots (CD63 and TSG101). Similar to previous work, there appear to be no change in ELV size, however, this study found that while low-intensity training elicited a ∼2-fold increase in EPC-derived ELVs, the moderate-intensity training increased release by ∼4-fold. Furthermore, EPC-derived ELVs from mice trained at moderate intensity were able to protect endothelial cells against hypoxia-induced apoptosis and angiogenic dysfunction *in vitro* to a greater extent to those derived from sedentary or low-intensity. This may support the notion that the training-induced increase in basal ELV number may be intensity-dependent, but also that the ELV cargo and bioactivity as well.

Work from Bertoldi et al. (2018) would suggest that the increase in circulating basal ELV in response to chronic exercise training occurs with very little training volume, and is observed with aging. For a 2-week training period, young (∼3 months), old (∼21 months), and very old (∼26 months) Wistar rats were allocated into sedentary or exercise groups. The animals exercised at ∼60% of their maximal oxygen uptake VO_2_ for 20 min⋅d^–1^, 7 d⋅wk^–1^. Following the last exercise bout, the animals were decapitated at either 1 h (to capture the acute exercise response) or 18 h post-exercise recovery, with the trunk blood collected and serum separated. EVs were isolated and characterized by miRCURY^TM^ Exosome Isolation Kit, with subsequent verification with NTA, CD63 levels by ELISA and acetylcholinesterase assay. In response to chronic exercise training, animals in all three age groups showed a significantly higher CD63 expression as compared to untrained animals (assessed at 18 h following last exercise bout). In line with other exercise data, there was no difference in CD63 expression in sedentary animals as compared to those acutely exercised. Interestingly, aged groups showed lower CD63 content as compared to young, regardless of their training status, which the authors suggested indicated lower circulating ELVs with aging. It is possible, however, that any temporal alterations in ELV content subsequent to an acute bout of exercise (i.e., that older individuals may not exhibit the same robust ELV response as younger counterparts) may be confounding any differences between age groups. Taken together, it would appear that chronic exercise leads to a higher level of basal circulating ELV content. The small number of studies, different blood preparations and the myriad of different ELV isolation techniques make firm conclusions difficult. To our knowledge, no chronic exercise training study focusing specifically on muscle-derived ELV content has been undertaken in humans. Future training studies, whether in murine models or humans, should aim to follow ISEV guidelines, minimize the impact from the ‘final bout’ of exercise training, and stringently verify the identity of the EV subpopulation under investigation.

### Exercise-Induced Modulations of miRNA Cargo

Given the extensive variations in both methodological approaches as well as observations of the acute ELVs response to a single exercise bout and/or the long-term adaptations to chronic exercise training, it is challenging to draw overarching conclusions. There appears to be an *inconsistent* but observable increase in blood borne ELV-related protein content in response to exercise (refer to Figure 2), though many factors appear to influence this observed change (e.g., exercise intensity, training status, and exercise modality). In line with this, there is similarly inconclusive evidence regarding alterations in ELV cargo, specifically miRNA, in response to exercise (Supplementary Table 1). Guescini et al. (2015) examined the relationship between VO_2max_ (determined via graded running test and verification phase on treadmill) and baseline expression of muscle related miRNA. Following ELV-enrichment from blood plasma via DC, filtration and UC, the study found the VO_2max_ was significantly correlated to presumed ‘myomiRs’ miR-1, miR-206, miR-499, and miR-133b. Further, this study also examined a subset of these participants that had a higher relative VO_2max_ as compared to the overall study cohort. Analysis of this subset revealed that only the expression of miR-181a-5p was significantly elevated following a bout of endurance exercise (45 min at ∼65% of VO_2max_); whereas, all other miRNA abundance was statistically unchanged 1 h following exercise cessation (Guescini et al., 2015). In contrast, a single bout of flywheel-based iso-inertial resistance exercise performed by recreationally trained men (5 sets by 10 reps) resulted in the increased abundance of some (miR-206 and miR-146a) but no other (miR-133b and miR-126-3p) miRNA scripts in UC-derived ELVs 2 h following exercise (Annibalini et al., 2019). miR-1-3p appears to be downregulated following an eccentric-contraction induced damage protocol (i.e., downhill running and plyometric jumping) as long as 24 h post-exercise (Lovett et al., 2018), underscoring that there may be selective packaging of miRNA during the response to exercise. Training status and/or health status appears to influence miRNA abundance in ELVs as well. Nair et al. (2020) compared healthy, recreationally trained (∼34 mL⋅min^–1^⋅kg^–1^ VO_2max_) to their sedentary (∼21 mL⋅min^–1^⋅kg^–1^ VO_2max_) counterparts, and observed that regular exercise increase resting expression of miR-486-5p, miR-215-5p, miR-941, while down-regulating miR-151b in UC, PEG-derived ELVs. Interestingly, in response to a single bout of exercise (6 min warm-up followed by cycling at 70% heart rate reserve for 40 min), the recreationally active group had a distinct profile of miRNA abundance in ELVs in response to the acute bout. Just as the ELV-related protein changes in response to exercise appear to be dependent on a variety of factors, it would appear the miRNA abundances follow similar patterns, with the immediately post-exercise collection period resulting in altered miRNA expression as that found 3 h post-exercise cessation (Nair et al., 2020). The notion that chronic exercise training modulates miRNA expression in ELV-enriched plasma and/or serum in basal conditions is highlighted by training studies performed in mouse and rat models utilizing different exercise training modalities (Chaturvedi et al., 2015; Hou et al., 2019; Yin et al., 2019) (Supplementary Table 1). While the physiological implications of exercise-induced alterations in the expression miRNA are outside the scope of this review, they will continued to be examined as key regulators of muscle-related biological processes such as atrophy (Hudson et al., 2014a) and hypertrophy (Fry et al., 2017).

Importantly, results from studies examining miRNA abundance following singe bouts and/or exercise training in ELV-enriched fractions without verifying size, concentration (e.g., NTA), morphological characteristics (i.e., EM) or markers of ELVs (e.g., TSG101 and ALIX) must be considered carefully. Without considering ELV isolation methodology, it is challenging to conclude whether the modulated miRNA signature following exercise is truly encapsulated in ELVs. Indeed, in a large number of studies, the term exosome has been inappropriately applied to describe EVs of a small size, isolated predominately through the use of differential centrifugation and high speed ultracentrifugation (Lotvall et al., 2014).

## Methodological Limitations and Framework for Studies on Exosomes and Exercise

First, it is a challenging task to study specific EV sub-populations in isolation of others. For example, the dynamic nature of exercise-mediated MV release, and the significant size-overlap between MVs and ELVs partially ‘masks’ our understanding of exercise effects on exosomes. As such, it is clear that there is still much to learn in determining the response of the exosome or ‘exosome-like’ EVs, as they may not always reflect the larger particles of circulating MVs (e.g., EMV, PMV, or SkMEVs). To highlight this discrepancy, Frühbeis et al. (2015) found that the MV population remained relatively unchanged in response to a bout of exhaustive cycling and/or running exercise; however, the subpopulation of EVs most likely to be exosomes (e.g., ∼100–130 nm in size, protein characteristics) showed a nearly twofold increase immediately after exercise cessation (Frühbeis et al., 2015). Therefore, while important work has been done examining the changes in the MV/microparticle subpopulations of EVs, the interested physiologist must be wary of ascribing the systemic benefits of exercise *exclusively* to non-exosome EV populations (i.e., microvesicles and/or apoptotic bodies). This notion, then, naturally applies to ascribing the bioactive cargo to various subpopulations as well. Total plasma abundances of miRNA are likely not reflective of those found within ELVs (Cheng et al., 2014), especially when concerning responses to exercise (D’souza et al., 2018). Therefore, in order to understand the impact of exosomes in response to exercise in isolation from other EV subpopulations, stringent collection methods for isolating or extracting an ‘exosome-enriched’ fraction from biofluids becomes paramount.

In line with this, significant inter-study variation and inherent technical challenges to EV research exist, and thus generalizations of findings pertaining to ELV biogenesis and kinetics in response to exercise are difficult to make. These challenges are mainly rooted in methodological issues related to sample purity and yield, including (i) blood preparation, (ii) ELV isolation/purification, and (iii) sample verification/characterization, which have prompted the development of a defined set of methodological criteria by the International Society of Extracellular Vesicles (ISEV) (Théry et al., 2018). Additionally, inter-study variation also stems from differences in participant characteristics, exercise interventions, testing conditions, and the timing of blood sampling.

### Timing of Blood Collections

Although circulating ELVs appear to be elevated prior to the LT/AT (Frühbeis et al., 2015; Brahmer et al., 2019), significantly higher levels may be detected immediately following exercise (Brahmer et al., 2019). This may be reflective of the rate of EV release (rate of appearance; EV R_a_) versus uptake/clearance (rate of disappearance; EV R_d_), and thus are likely reflective of differences in training status (e.g., aerobic fitness and oxidative capacity). Evidence would suggest that the appearance of ELVs in circulation is relatively rapid (i.e., before an appreciable increase in exercise intensity), and therefore timing of blood sampling should be considered with this in mind. ELV content appears to be elevated immediately after a relatively short but intense bout of exercise (i.e., step incremental max test), likely returning to baseline at ∼1 h post-exercise (Frühbeis et al., 2015; Brahmer et al., 2019). The timing of blood draws should be taken into consideration as EV R_d_ may exceed EV R_a_ following a 45–90 min moderate-intensity continuous effort. Recent work examining the secretion and clearance of plasma SEC-derived ELVs would further underscore this notion. Using specific protein-labeled SEC-derived ELVs and pharmacokinetic analysis, Matsumoto et al. (2020) revealed a rapid rate of disappearance of intravenously administered ELVs, with a half-life of ∼7 min. These findings may reflect the relative transitory nature of exercise induced ELV appearance in circulation, and highlight the importance of collection timing.

### Blood Draw Techniques

To assess the effect of contractile activity on EVs and exercise factors in the absence of confounding pre-analytical factors, it is important to standardize the blood sampling technique, blood fraction of interest, and initial blood preparation steps (Lacroix et al., 2013; Witwer et al., 2013). The use of an appropriate size needle/catheter that minimizes agitation and/or rupture-risk of various blood cell populations (e.g., platelets, WBCs, and RBCs), if possible 21- or 22-g needle is preferred (Lacroix et al., 2012; Wisgrill et al., 2016). The prolonged use of tourniquets should also be avoided (Lippi et al., 2006), and the first 2–3 mL of blood discarded or used for other analytes if possible (Hefler et al., 2004; van Ierssel et al., 2010; Coumans et al., 2017).

### Sample Handling, Blood Fractions, and Anticoagulants

Once the blood has been drawn into an evacuated tube, the sample should be kept upright, handled gently, hemolytic effects noted (Pritchard et al., 2012) and then processed at RT as soon as possible; the total time between blood sampling and analysis or storage at −80°C should be minimized and standardized, as some blood fraction contaminants such as platelets may release EVs during fragmentation stemming from a freeze-thaw cycle (Lacroix et al., 2012; Mitchell et al., 2016). In line with this, the use of blood plasma is preferable over serum (Yuana et al., 2011; Coumans et al., 2017), as additional EVs or exosomes may be released during the process of clot formation (Wolf, 1967). However, if serum is the only option because of downstream analytical reasons (e.g., miRNA), an intermediate clotting time may be used (≥30 min ≤ 1 h at RT) (Cheng et al., 2014; Maffioletti et al., 2014; Andreu et al., 2016), but the effect of clotting on EVs/exercise factor release must be considered when interpreting the data. In the same line of reasoning, the use of platelet-free plasma (PFP) is obviously advantageous for mitigation of platelet-derived EVs, which is simply done by adding an extra, 15-min spin step (Lacroix et al., 2012, 2013; Wisgrill et al., 2016; Coumans et al., 2017). Platelet-free plasma (PFP) thus appears to be more representative of the acute exercise effect as compared to platelet-rich plasma (PRP) and serum, both of which are additionally reflective of the platelet pool and/or the clotting process. Considering that the choice of anti-coagulant may affect the release of EVs/exercise factors and potentially limit downstream analyses (Tsuchimine et al., 2014; Coumans et al., 2017), multiple types of blood preparation would be preferred. For EVs, several anticoagulants have been utilized, including but not limited to, ethylenediaminetetraacetic acid (EDTA), sodium fluoride, heparin and sodium citrate (Yuana et al., 2011; Lacroix et al., 2013; Witwer et al., 2013). However, whereas EDTA is suitable for RNA analysis (van Eijndhoven et al., 2016), heparin may interfere with subsequent polymerase chain reaction experimentation (Beutler et al., 1990). For EV isolations, sodium citrate may be the preferred choice over other anticoagulants (Lacroix et al., 2013), although more research is needed in this area, specifically for ELVs. Taken together, blood collection methodology must be considered carefully, and collection timing appears to be paramount for the accurate analysis of MV and ELV exercise kinetics.

### Isolation, Purification, Storage and Identification of ELVs

Peripheral blood contains a spectrum of EVs with molecular signatures and cargos reflective of the type, function, secretory capacity, and overall abundance of their cell origin(s). Thus, one of the most challenging aspects of ‘exosome’ research is that a vast majority of isolates will contain some degree of contamination and a diverse vesicle population, including various blood proteins (albumin, chylomicrons, and other lipoproteins), MVs, and ELVs. Some contaminants may overlap in size/diameter with ELVs and may also respond to exercise stimuli, which complicates delineation of the exercise response of ELVs. Isolation of specific vesicle sub-populations thereby comes with several methodological challenges, particularly related to the purification and identification of ELVs, which has led to strict procedural guidelines. In response to the growing interest in the field, ISEV put forth a series of guidelines that outlined the minimal requirements for the proper determination of exosomes in 2014 (Lotvall et al., 2014). These guidelines have helped to shape some of the more recent examinations of exosomes in response to exercise (Frühbeis et al., 2015; Lovett et al., 2018; Whitham et al., 2018; Brahmer et al., 2019). Since then, the MISEV2014 guidelines (although still valid) have been amended into ISEV2018 guidelines in attempts to outline what should be mandatory and encouraged for researchers partaking in EV science and research.

ISEV2018 guidelines recognize that there is no ‘optimal’ ELV isolation/enrichment method, the study of the response to exercise requires additional care be taken. As lipoproteins are moderately enhanced by acute exercise (Ferguson et al., 1998; Lira et al., 2010; Harrison et al., 2012; Wang and Xu, 2017), and particles such as chylomicrons overlap in size with ELVs, every effort should be made to minimize the presence of these contaminating factors prior to the analysis phase. As recently shown by Brahmer et al. (2019), this is particularly important if NTA is to be used as an indicator for the ELV response to exercise. To date, the most commonly used isolation and/or enrichment methods in the literature have been microfiltration, differential centrifugation/ultracentrifugation (DC/UC), and polyethylene glycol-based kits (PEG; ExoQuick^TM^ and TIER) (Baranyai et al., 2015; Wiklander et al., 2015; Rider et al., 2016; Weng et al., 2016). DC/UC applies a series of centrifugation steps with increasing rotor speeds (in excess of ∼100,000 g), which is used to progressively remove larger subfractions of EVs (Théry et al., 2006; Taylor and Shah, 2015) – the efficacy of which can be enhanced by diluting viscous fluids such as plasma (Théry et al., 2006). The isolation or enrichment of exosomes is complicated by overlapping size and density profiles (Yáñez-Mó et al., 2015) paired with the notion that there may be multiple exosome subpopulations (Yang and Gould, 2013). Unfortunately, these methods are time-consuming, require expensive equipment (DC/UC), and/or yield significant contamination (DC/UC and PEG) (Coumans et al., 2017). Although microfiltration may aid in removing larger vesicles, such as apoptotic bodies and MVs, prior high-speed centrifugation and PEGylation, significant contamination still remains in human samples. Conversely, density-gradient DC/UC (e.g., Optiprep, sucrose gradient), size-exclusion chromatography (SEC), and immunoaffinity capture are *less* commonly used in exercise studies [with the exception of Brahmer et al. (2019)] but have the distinct advantage of yielding more pure isolates (Coumans et al., 2017). Despite the opportunity to minimize contaminating particles, and the downstream functionality of ELVs isolated via SEC as compared to other precipitating agents (Gámez-Valero et al., 2016; Mol et al., 2017), there are still limitations of the methodology (Simonsen, 2017; Burgess, 2018). Indeed, while the use of SEC has yielded relatively pure ELV preparations (Böing et al., 2014; Lobb et al., 2015; Welton et al., 2015), vesicles that overlap in size will be isolated together, making it challenging to separate ELVs from chylomicrons and very low-density lipoproteins (VLDL) (Brennan et al., 2020). Furthermore, the purity of ELV-enriched fractions, separation of ELV markers from soluble contaminants, and the efficiency of ELV isolation may vary depending on the SEC column used (Baranyai et al., 2015). Finally, given the restrictive process, SEC may also result in low vesicle yield, albeit with a greater purity compared to other methods. As with any developing methodology, a combination of methods (e.g., SEC followed by UC) will continue to be utilized and ultimately result in improvements over approaches that rely on single methods (Wei et al., 2020), though SEC-enriched ELVs remain an excellent methodological approach.

Ideally, all enrichment and downstream analyses should be performed on fresh, citrated PFP and ELVs; however, there appear to be no significant differences in morphology, marker expression, or particle characteristics (e.g., diameter or particle concentration) following isolates being snap frozen in liquid nitrogen (Yuana et al., 2011) and stored at −80°C. Previous work would suggest that plasma-derived EVs and ELVs appear to be stable amid freeze-thaw cycles in some conditions (Lacroix et al., 2012). Indeed, up to three freeze-thaw cycles of PFP had no effect on MV counts (Jayachandran et al., 2012). While the size of ELV decreased when stored at 4 and 37°C, multiple freezing to −20°C and thawing did not affect the ELV size as assessed by NTA and scanning electron microscopy, ultimately suggesting structural preservation (Sokolova et al., 2011). Whether this protection extends to the contents of ELVs, remains to be elucidated. Work has shown that the biological activities of ELVs (Lorincz et al., 2014) or degradation of ELV-contained RNA (Wu et al., 2015) may occur over long term storage at −80°C.

For more in-depth reviews of various exosome isolation methodologies, the reader is encouraged to refer to previous literature (Théry et al., 2006; Taylor and Shah, 2015; Yáñez-Mó et al., 2015; Coumans et al., 2017). Ultimately, separation of non-vesicular entities from ELVs is not completely (i.e., 100%) achieved by common isolation protocols, particularly centrifugation-based methods (Théry et al., 2018), and thus the confirmation of an enrichment of ELV is required.

Regardless of the isolation methodology of choice, it is imperative to adhere to the ISEV guidelines for EV characterization in an attempt to minimize variation between studies and improve the interpretability of the results. Semi-quantitative methods may be most helpful when characterizing the ELV response to exercise (Brahmer et al., 2019) because a number of contaminating factors may interfere with NTA profiles and electron microscopy (e.g., lipoproteins, chylomicrons, and other blood proteins). The utilization of protein markers for CD9, CD63, CD81, TSG101, and ALIX for positive confirmation of ELVs, and the use of ApoA1, albumin, and calnexin as markers of contamination (i.e, a negative control) would be appropriate (Lotvall et al., 2014; Théry et al., 2018). The challenges in characterization of ELVs can be, in part, overcome by utilizing several methodologies, including immunoblotting, NTA and electron microscopy.

### Participant Characteristics, Nutritional State, and Exercise Design

In order to elucidate the physiological response(s) to exercise, it is recommended to study a homogenous population and control for the major, inter-individual participant characteristics that affect human biology. Given the state of the field of exercise physiology, considerations must be made for age, gender, disease state and/or musculoskeletal injury, medication use, nutritional state (i.e., fasted or fed), and training status to ensure the internal validity of the study. Other variables with apparent biological effects in exercising humans are nutritional intake (fasting ∼8–10 h; no caffeine, alcohol, or smoking), time since last exercise session (no physical activity > 24 h), time of testing, testing conditions (e.g., humidity and temperature), length of warm-up and the exercise program design (mode, intensity, and duration). Additional variables that may affect the EV response to exercise are sleep and hydration status, both known to affect human performance in general. While the effects of external cues, such as the number or gender of technicians, verbal encouragement, and music playing during the test(s), are not known, these variables should be controlled for as well. To further improve the reproducibility between studies on the effects of exercise on EVs, we therefore suggest that that these factors should be included as a minimal requirement in the methods sections of future publications in the area of exercise science.

## Conclusion

The findings that EVs and ELVs can facilitate intracellular communication through the delivery of cargo marks an exciting new development in the field of metabolism and exercise physiology. The capabilities of circulating ELVs to facilitate tissue crosstalk may represent a novel mechanism underlying the multi-systemic benefits of exercise. Many challenges remain surrounding the isolation of ELVs, identification of tissue of origin, and the response to exercise, however, the opportunity to understand how exercising skeletal muscle can promote whole-body health is an exciting one.

## Author Contributions

JPN: drafting, manuscript writing, and final approval of the manuscript. GW, AD, MIN, and MAT: manuscript writing and final approval of the manuscript. All authors contributed to the article and approved the submitted version.

## Conflict of Interest

Exerkine Corporation is a biotechnology company that develops and commercializes therapies based on nutritional supplements, exercise-derived factors (‘exerkines’), and extracellular vesicles to treat and diagnose genetic disorders, chronic diseases, and aging. MAT is the founder, CEO, and CSO of Exerkine Corporation, which provided support in the form of salary to MIN. MAT and MIN are also shareholders in the company. The funders had no additional roles in the decision to publish or preparation of the manuscript. The remaining authors declare that the research was conducted in the absence of any commercial or financial relationships that could be construed as a potential conflict of interest.
