# Supplementary material for: Extracellular Vesicles and Exosomes: Insights From Exercise Science
Source: Front Physiol. 2021 Feb 1;11:604274. doi: 10.3389/fphys.2020.604274 (PMC7882633; doi:10.3389/fphys.2020.604274)
Supplement: Supplementary file 1 [file Table_1.pdf]

Publication	Exercise Protocol	Bio-fluid	Author-used Terminology	Isolation Method	miRNA Methodology	miRNA Species	Collection Time Periods miRNA results		
**ACUTE EXERCISE STIMULUS**									
							2h Post-Ex	24h Post-Ex	
Lovett et al., 2018	PMJ (10 x 10 maximal jumps) followed by DHR (5 x 4 min DHR at 10km/h at 10% decline) in untrained males	PLA	EVs in exosome size range	DC<br>SEC	TaqMan Advanced miRNA assays	miR-1	↔	↔	
						miR-31	↑	↑	
						miR-133a	↔	↔	
						miR-133b	↔	↔	
						miR-206	ND	ND	
						miR-208b	↔	↔	
						miR-486	↔	↔	
						miR-499a	ND	ND	
							Post-Ex	4h Post-Ex	
D'Souza et al., 2018	10 x 60s intervals of cycling at peak power output in healthy men	PLA	Exosomes	DC<br>SEC<br>0.22 µm filter	TaqMan Advanced miRNA assays (*n* = 29, not all listed)	miR-1-3p	↑	↔	
						miR-16-5p	↑	↔	
						miR-23a-3p	↑	↔	
						miR-23b-3p	↑	↔	
						miR-126-3p	↑	↔	
						miR-150-5p	↑	↔	
						miR-186-5p	↑	↔	
						miR-208a-3p	↑	↔	
						miR-222-3p	↑	↔	
						miR-378a-5p	↑	↔	
						miR-451a	↑	↔	
						miR-486-5p	↑	↔	
						Not Detectable (ND)			
						miR-423-5p	miR-133a-3p		
						miR-149-5p	miR-191-5p		
						miR-494-3p	miR-206		
						miR-24-1-5p	miR-320a		
						miR-27a-5p	miR-361-5p		
						miR-33a-3p	miR-499a-3p		
							Post-Ex		
Guescini et al., 2015	40 min vigorous intensity treadmill running (15 min warm-up followed by 40 min at ~80% VO_{2max} at 1% grade) in well-trained men	PLA	EVs	DC<br>0.22 um filter<br>UC<br>IBI	TaqMan Advanced miRNA assays	miR-1	↔		
						miR-24	↔		
						miR-133a	↔		
						miR-133b	↔		
						miR-136a	↔		
						miR-181a-5p	↑		
						miR-206	↔		
						miR-499	↔		
							Post-Ex	2h Post-Ex	48h Post-Ex
Annibalini et al., 2019	Resistance training (5 x 10 maximal squats, 3 min rest interval) on a flywheel in trained men	PLA	EVs	DC<br>UC	miRCURY LNA miRNA PCR Assay	miR-126-3p	↔	↔	↔
						miR-133b	↔	↔	↔
						miR-146a-5p	↑	↔	↔
						miR-206	↑	↔	↔

Publication	Exercise Protocol	Bio-fluid	Author-used Terminology	Isolation Method	miRNA Methodology	miRNA Species	Collection Time Periods miRNA results		
**ACUTE EXERCISE STIMULUS (continued)**									
							Post-Ex		
Oliveira et al., 2018	Treadmill 40 min at low intensity (14-16 m·min^{-1}, ~20% below MLSS) in untrained Wistar rats.<br>Treadmill 40 min at moderate intensity (20-22 m·min^{-1}, on MLSS)<br>Treadmill 40 min at high intensity (24-26 m·min^{-1}, 20% above MLSS)	SER	Exosome-like small EVs	DC<br>Exoquick^{TM}<br>Precipitation	Small RNA library (NextGen) (*n* = 765 mature miRNAs of *R. norvegicus* miRBase database)<br><br>All intensities collapsed for analysis.	miR-25-3p	↓		
						miR-93-5p	↓		
						miR-103-3p	↓		
						miR-128-3P	↓		
						miR-142-5P	↓		
						miR-148a-3p	↓		
						miR-191a-5p	↓		
						miR-3068-3p	↓		
						miR-10b-5p	↑		
						miR-142-3p	↑		
						miR-330-5p	↑		
						miR-410-3p	↑		
							Post-Ex	1h Post-Ex	48h Post-Ex
Yin et al., 2019	UH running (20 m·min^{-1} at a 15% grade for 90 min) in male Sprague-Dawley rats	PLA	Exosomes	DC<br>Exoquick^{TM}<br>Precipitation	Custom-design (synthetic miRNA oligonucleotides)	miR-1	↔	↔	↔
						miR-133a	↔	↔	↔
						miR-133b	↔	↔	↔
						miR-206	↔	↔	↔
	.					miR-208	↔	↔	↔
						miR-499	↔	↔	↔
	DH running (20 m·min^{-1} at a 15% grade for 90 min) in male Sprague-Dawley rats					miR-1	↑	↑	↔
						miR-133a	↑	↔	↔
						miR-133b	↑	↔	↔
						miR-206	↑	↔	↔
						miR-208	↑	↑	↔
						miR-499	↑	↔	↔
							Post-Ex		3h Post-Ex
Nair et al., 2020	Cycling exercise for 40 min at ~70% HRR in recreationally trained (RT; ~34mL·min^{-1}·kg^{-1}) and sedentary (SED; ~21 mL·min^{-1}·kg^{-1})	PLA	Exosomes	DC<br>Exoquick^{TM}<br>Precipitation<br>UC	Small RNA Library Sequencing	<u>RT Group</u>			
						miR-383-5p	↑	miR-34b-3p	↑
						miR-339-5p	↑	miR-129-2-3p	↑
						miR-74-3p	↑	miR-138-1-3p	↑
						miR-486-5p	↓	miR-671-3p	↑
						miR-148a-p	↓	miR-885-5p	↑
						let-7b-5p	↓	miR-486-5p	↓
						miR-206	↓	miR-629-5p	↓
								miR-16-2-3p	↓
						<u>SED Group</u>			
						miR4433b-3p	↓	miR4433b-3p	↓
						miR-505-3p	↑	mir-378c	↓
						miR-29b-3p	↑	miR-151b	↓
						miR-203a-3p	↑	miR-151a-5p	↓
						miR-384	↑	hsa-miR-151b	↓
						miR-451a	↑		
						miR223-3p	↑		
						miR-218-5p	↑		
						miR-495-3p	↑		

Publication	Exercise Protocol	Bio-fluid	Author-used Terminology	Isolation Method	miRNA Methodology	miRNA Species	Collection Time Periods miRNA results
**CHRONIC EXERCISE TRAINING**							
							Following Training
Chaturvedi et al., 2015	8-wk treadmill training in db/+ mice (7 m·min^{-1}) and db/db mice (10 m·min^{-1}) for 300m/d, 5d/wk	SER	Exosomes	DC<br>0.2 µm filter<br>UC	Custom design (synthetic miRNA oligonucleotides)	miR-455<br>miR-29b<br>miR-323-5p<br>miR-466	↑<br>↑<br>↔<br>↔
							Following Training
Hou et al., 2019	4-wk swimming training (10m progressed to 90m/day ~7days/wk) in Sprague-Dawley rats	PLA	Exosomes	DC<br>Exoquick^{TM}<br>Precipitation<br>UC	miRNA library sequencing (Commercial service)	miR-3571<br>miR-1-3p<br>miR-342-5p<br>miR-122-5p<br>miR-196b-5p<br>miR-486<br>miR-208-3p<br>miR-3591<br>miR-184<br>miR-760-3p<br>miR-99a-5p<br>miR-191-5p<br>miR-494-3p<br>miR-206-3p	↑<br>↑<br>↑<br>↑<br>↑<br>↑<br>↑<br>↑<br>↑<br>↑<br>↑<br>↓<br>↔<br>↔

Supplementary Table 1: The effect of acute or chronic exercise on miRNA abundance in ELV-enriched blood borne fractions. DC; differential centrifugation; DHR: downhill running; EM: electron microscopy; EV: Extracellular Vesicles; IBI; Immuno-bead isolation; PLA; Plasma; PMJ: plyometric jumping; RT; recreationally trained prior to study initiation, SEC: Size Exclusion Chromatography; SER: serum; UC: ultracentrifugation; UHR: uphill running; ↑; statistically significant increase, ↔ non-significant changes, ↓; significant decrease denoted by statistical tests performed in the publication of origin.
